# An Integrated Hypothesis on the Domestication of *Bactris gasipaes*

**DOI:** 10.1371/journal.pone.0144644

**Published:** 2015-12-10

**Authors:** Gea Galluzzi, Dominique Dufour, Evert Thomas, Maarten van Zonneveld, Andrés Felipe Escobar Salamanca, Andrés Giraldo Toro, Andrés Rivera, Hector Salazar Duque, Harold Suárez Baron, Gerardo Gallego, Xavier Scheldeman, Alonso Gonzalez Mejia

**Affiliations:** 1 Regional Office for the Americas, Bioversity International, Cali, Valle del Cauca, Colombia; 2 CIRAD, Centro de cooperación internacional en investigación agronómica para el desarrollo, Cali, Valle del Cauca, Colombia; 3 Sub-regional Office for the Americas, Bioversity International, Turrialba, Cartago,Costa Rica; 4 CIAT, Centro Internacional de Agricultura Tropical, Cali, Valle del Cauca, Colombia; 5 Programa de Biología, Universidad del Quindío, Armenia, Colombia; 6 Instituto de Biología, Universidad de Antioquia, Medellin, Colombia; University of Florida, UNITED STATES

## Abstract

Peach palm (*Bactris gasipaes* Kunth) has had a central place in the livelihoods of people in the Americas since pre-Columbian times, notably for its edible fruits and multi-purpose wood. The botanical taxon includes both domesticated and wild varieties. Domesticated var *gasipaes* is believed to derive from one or more of the three wild types of var. *chichagui* identified today, although the exact dynamics and location of the domestication are still uncertain. Drawing on a combination of molecular and phenotypic diversity data, modeling of past climate suitability and existing literature, we present an integrated hypothesis about peach palm’s domestication. We support a single initial domestication event in south western Amazonia, giving rise to var. *chichagui* type 3, the putative incipient domesticate. We argue that subsequent dispersal by humans across western Amazonia, and possibly into Central America allowed for secondary domestication events through hybridization with resident wild populations, and differential human selection pressures, resulting in the diversity of present-day landraces. The high phenotypic diversity in the Ecuadorian and northern Peruvian Amazon suggest that human selection of different traits was particularly intense there. While acknowledging the need for further data collection, we believe that our results contribute new insights and tools to understand domestication and dispersal patterns of this important native staple, as well as to plan for its conservation.

## Introduction

**Fig 1:**
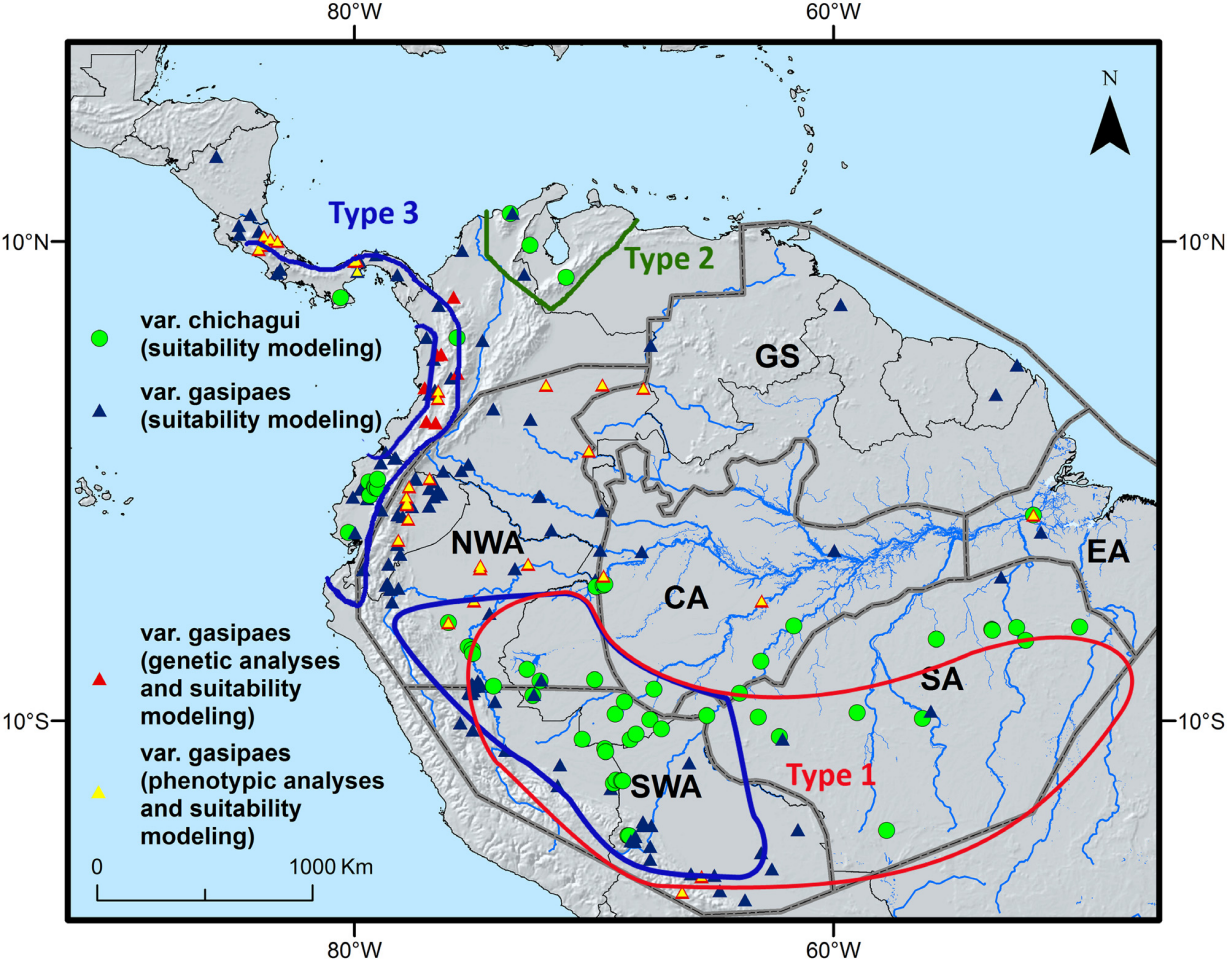
The distribution of cultivated (*Bactris gasipaes* var. *gasipaes*, triangles) and wild (var. *chichagui*, dots) peach palm observation points. The points used only for modelling and gathered from sources other than this study are reported in blue and green, whereas the points referring to samples used in this study are shown in red or yellow, depending on the additional analyses carried out on each (genetic or phenotypic characterization). The red, blue and green polygons show the approximate distribution of *Bactris* wild types [20]; the grey lines divide the different regions of Amazonia (NWA: north-western Amazonia, GS: Guyana shield, EA: eastern Amazonia, CA: central Amazonia, SA: southern Amazonia, SWA: south-western Amazonia; after [34]).

Peach palm (*Bactris gasipaes* Kunth) is the only palm species with domesticated populations in the Neotropics [1]. It is an alogamous, monoecious palm tree which adapts well to a broad range of ecological conditions, but prefers well-drained deep soils at altitudes below 800 m.a.s.l., with annual precipitation between 2,000–5,000 mm and annual mean temperature above 24°C [2]. Peach palm is widely distributed across the neotropics; outside of commercial plantations, in homegardens and orchards, it is found from Honduras to Bolivia. The species’ starchy fruit has been an important staple in numerous lowland societies since pre-Columbian times, whereas its hard and elastic wood is used for construction purposes and a plethora of handicrafts and hunting and fighting gears [3,4]. The most recent revision of the *Bactris* genus [5] gathered all cultivated populations of peach palm into var. *gasipaes* and all wild populations (previously identified as different species) into var. *chichagui*. Within var. *chichagui* three types were proposed, based on fruit morphological traits [6]: types 1 and 2 have very small fruits (1–2 g) and occur in southern and southwestern Amazonia, and northern South America, respectively [7,8]; type 3 has small fruits (3 to 10 g, rarely 15 g) and a disjunctive distribution, from southwestern to western Amazonia, and from coastal Ecuador over western Colombia to southern Central America [9] (Fig 1). Several authors have argued that var. *gasipaes* may be derived from var. *chichagui* type 3, possibly through hybridization with type 1 [8–11] but with no definitive indication of when and where this hybridization, and subsequent domestication, may have taken place.

The earliest archaeological evidence of crop domestication in South America is from western Ecuador 12,000 years ago, and concerns a now abandoned squash species [12]. The earliest Amazonian site with remains unmistakingly pointing to the use of palm species by ancient humans is in Pedra Pintada, in Monte Alegre (Pará, Brazil) dated to >11,200 years ago [13]. Evidence for palm remains is particularly widespread after 9,000 BP, such as in the Amazon along the Colombian Caquetá river [14,15]. Although no remains of peach palm were found at the oldest sites, it is clear that it was a fully fledged crop long before the arrival of Europeans in the New World. Yet, as many Amazonian crops, it has maintained a range of populations and types stemming the wild-semidomesticated-domesticated continuum [16].

Three main hypotheses about the locations of peach palm domestication exist. Patiño Rodriguez [3] pointed to an initial domestication west of the Andes, which is supported by archaeological findings in the Pacific lowlands of Colombia, close to the Cauca river valley, where wild type 3 is found [15]. A second hypothesis proposed that cultivated peach palm is the result of several independent domestication processes by different lowland societies, possibly on both sides of the Andes [8,17]. According to a third hypothesis, which is supported by the majority of authors, southwestern Amazonia would be the most likely site of early peach palm domestication, from which human-mediated dispersal subsequently occurred [8–10,18–21]. This hypothesis has recently been confirmed by the findings of a chloroplast DNA marker study [9]. There is little support for a central American origin. Most authors have defended a principally northward migration on the basis of the low levels of diversity usually observed in Central American materials [21,22]. It is likely that peach palm was introduced to Central America only around 2,300 to 1,700 years ago [23], as a staple food by the pre-Columbian Chibcha civilization from South America [24], an event which would have been associated with a genetic bottleneck.

Drawing on the results of a genetic and morpho-biochemical characterization of a continent-wide peach palm collection, the purpose of this study is to contribute to the debate on the origin and dispersal of peach palm in the neotropics. We explore the possibility of a fourth hypothesis, being the conjunction of the second and third hypotheses mentioned above; more specifically, we suggest a primary domestication event in southwestern Amazonia followed by secondary domestication at different sites of peach palm’s current distribution coinciding with the location of some of the currently described landraces. Hernandez-Ugalde *et al* [8] argued that the distribution of genetic diversity in peach palm is the combined outcome of the species’ natural history, the great variety of habitat conditions across its distribution range, prolongued isolation of populations in glacial refugia, and the influence of human domestication, among others. On the assumption that prolongued isolation and human management are the two main factors that may have influenced the distribution of the neutral genetic diversity we measured, we reconstructed the distribution of suitable habitats of peach palm’s ancestral populations during the Last Glacial Maximum (LGM), occurring between 26,500 and 19,000–20,000 years ago [25], which—as is well known- left a profound impact on the vegetation in the Amazon basin [26]. We postulate that collection sites high in genetic diversity located close to, or within areas with suitable LGM habitats might be indicative of secondary domestication processes accompanied by hybridization events with resident wild populations (cf. [8]), and that high diversity in phenotypic traits is indicative of a long history of human management and use [20,27]. Based on our findings, we present a hypothesis of the domestication history of peach palm that might explain the dynamics behind the emergence of the currently known, disjuntly distributed landraces across Latin America from an incipient domestication event in southwestern Amazonia. We use the patterns observed in neutral genetic diversity and variation in morphological and biochemical fruit traits, to identify priority areas for on-farm conservation of peach palm, also considering the potential impacts of anthropogenic climate change on the species’ future distribution.

## Materials and Methods

### Genetic and phenotypic data

Leaf material was collected from 87 individual plants coming from two important *ex situ* collections, i.e. that of CATIE (Centro Agronómico Tropical de Investigación y Enseñanza), in Turrialba, Costa Rica and that of INIA (Instituto Nacional de Investigación Agraria) in Iquitos, Peru, as well as from collections in commercial farms in Colombia; samples were obtained/collected between 2009 and 2010. Material transfer agreements were signed with the genebanks of CATIE and INIA for their respective samples and permission was obtained by the owners of the farms from which the samples from Colombia were collected; only domesticated specimens were collected on-farm.

A list of the material and passport information is given in S1 Table. The genebank samples (one tree per accession) were chosen in order to represent the broad phenotypic diversity available in both collections. Based on measures of fruit weight, three accessions in the dataset appeared to be from wild populations (fruit weight < 10 g), possibly belonging to *B*. *gasipaes* var. *chichagui*. These individuals (marked with an asterisk in S1 Table) all come from the Amazon basin, and were removed from the analyses.

Collected leaf material was dried and conserved in zip-lock plastic bags with silica gel until DNA extraction (see S1 Text for details). Peach-palm specific microsatellite markers [28] (see S2 Table for primer sequences) were amplified on the samples.

Population genetic parameters such as allele number, average allele frequency, heterozygosity (expected and observed), and the interindividual fixation index (Fis), were calculated and a Mantel test was conducted between the samples’ geographical distances and their genetic distances [29], searching for evidence of genetic structuring and patterns of isolation by distance. All analyses were performed in R [30], using packages Adegenet 1.4–2 [31] and Vegan 2.0–8 [32].

Morphological and biochemical traits were measured (see S2 Text for details) on a subset of the molecular dataset, which includes the 67 cultivated accessions corresponding to those from CATIE and INIA Peru but excludes the samples collected on-farm in Colombia.

### Suitability modeling

We constructed a dataset containing 221 presence points of which 166 pertained to the cultivated form (*B*. *gasipaes* var. *gasipaes*) and 55 to the wild form (*B*. *gasiapes* var. *chichagui*). Additional presence points to those of the present study were collected from a variety of sources, including published papers [6,8], members of the Latin American Forest Genetic Resources Network (Laforgen), and the Global Biodiversity Information Facility [33]. The full dataset is shown in Fig 1, with different colours and symbols distinguishing wild from cultivated samples, as well as samples used only for modelling from those on which genetic and/or phenotypic diversity analyses were carried out.

We characterized the spatial distribution of favorable habitat conditions of peach palm under current, past, and future climatic conditions by means of suitability mapping based on ensembles of modelling algorithms, implemented in R package BiodiversityR [35] (see S3 Text for details on the modelling algorithms considered and calibration methods).

We evaluated the ability of all individual modeling algorithms to cope with spatial autocorrelation by calculating calibrated Area Under Curve (cAUC) values and comparing these with a geographical null model [36]. To this end, we (i) randomly partitioned both presence and background points in five groups, (ii) carried out five rounds of calibrating and testing for all models (including the geographical null model), each time using four partitions for model calibration, and one partition for model testing from which spatial sorting bias was removed [36]. We repeated this process twice and compared the ten resulting cAUCs of each of the distribution models with the ten cAUCs of the geographical null model by means of Mann-Whitney tests. Only models that gave AUC values that were significantly higher than the null model (p<0.05) were retained in the ensemble model used for projections. In a next step, we calculated the cAUC values for all possible ensemble combinations resulting from the retained models. Each ensemble combination was constructed as the weighted average of its individual composing models, using the cAUC values as weights. The respective ensembles with the highest cAUC scores were used for projection to past and future climates.

**Fig 2:**
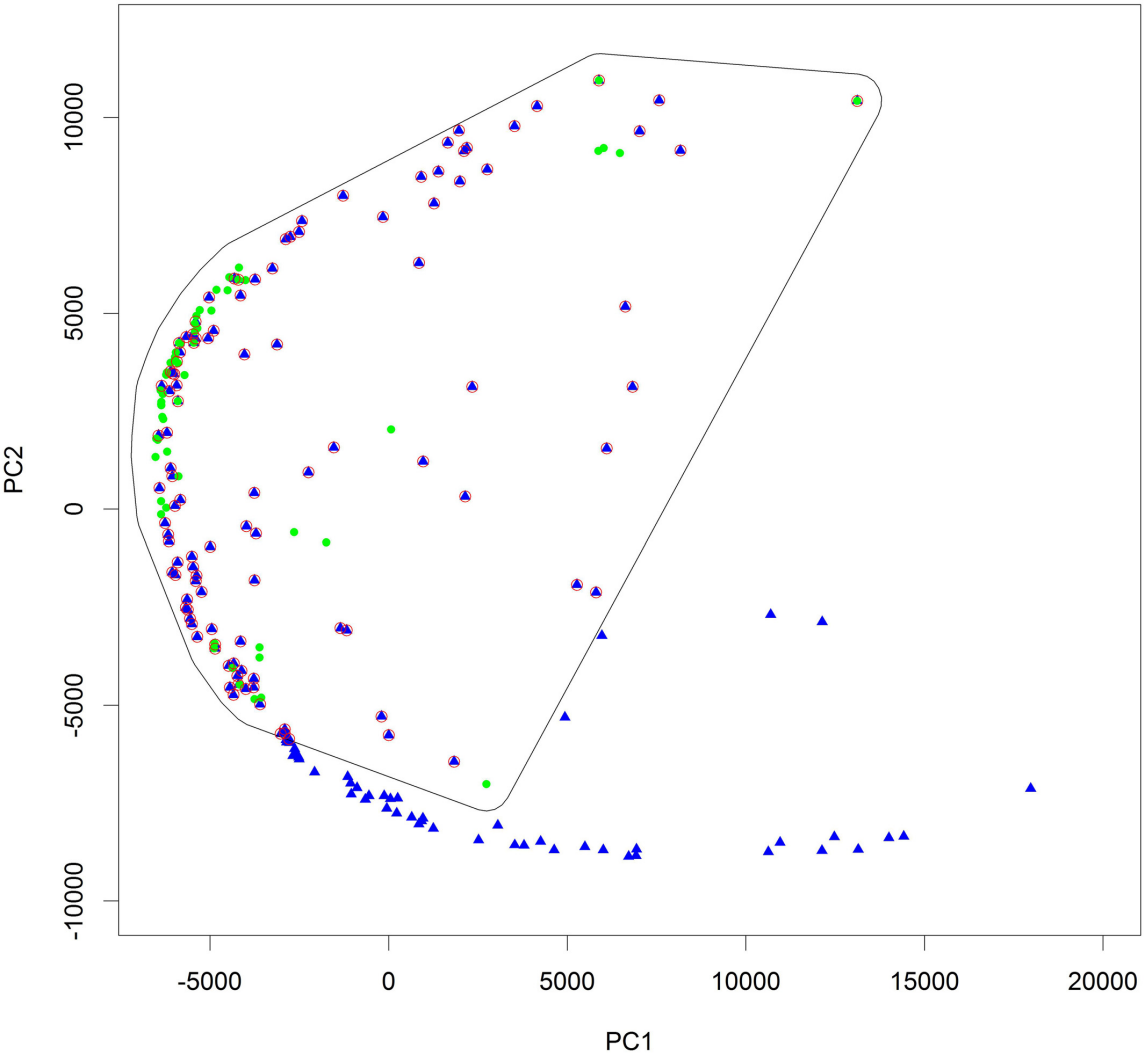
Principal component analysis of peach palm wild (var. *chichagui*) and domesticated (var. *gasipaes*) populations. As in Fig 1, green poins are the wild form (var. *chichagui*) and blue triangles are the cultivated form (var. *gasipaes*). The two components shown here explain 94% of the overall variation in the data. The polygon represents the convex hull area constructed around all var. *chichagui* points, extended with a 3% buffer of the largest hull axis.

Removal of spatial sorting bias in testing data for different model calibrations normally yields average cAUC values for the null model of approximately 0.5 which is equivalent to a random draw [36]. However, model calibration based on only wild points for projection to the LGM yielded an average AUC value of 0.58 for the null model, with none of the tested distribution models performing significantly better. This means that none of the models was able to yield a better suitability prediction than the suitability estimates based on inverse geographical distance from presence points provided by the nullmodel, hence seriously limiting the models’ predictive power. As this situation was most likely due to the relatively limited number of presence points for the overall range of the wild type, and the relatively clustered occurrence of these points, we considered two scenarios. In a first scenario we used only Maxent for being the model with the highest average cAUC value of all models (0.6). In a second scenario, keeping in mind that cultivated and wild peach palm are genetically very close, being variants of the same botanical species, and have a high tendency to hybridize [37,38], we extended the set of presence points from the wild form with those of the cultivated form pertaining to the same ecological niche as the wild points. The purpose of the latter procedure was to exclude those cultivated trees occurring in areas beyond the known niche of the wild variants, which may be the consequence of changed adaptive capacity resulting from human selection, and hence could bias the detection of suitable areas during the LGM. To eliminate such occurrences, we delimited the ecological niche of var. *chichagui* by drawing a convex hull, extended with a 3% buffer of the largest hull axis, around all presence points in the environmental space determined by a the two first axes of a PCA based on the bioclimatic variables (Fig 2). All points falling within this extended convex hull area and pertaining to var. *gasipaes* were considered to occur in the ecological niche occupied by var. *chichagui*, based on the presence points here considered. We evaluated the niche overlap between the presence points used in both scenarios based on the framework proposed by Broennimann *et al*[39], whereby niche overlap is measured in a gridded and smoothed PCA-conditioned environmental space, through the use of Schoener’s D which varies from 0 (no overlap) to 1 (complete overlap). A comparison of the overlap between the niches occupied by presence points considered in both scenarios showed a Schoener’s D of 0.7, and statistically significant niche equivalencies and similarities (at 0.05 treshold), justifying the validity of our second scenario. Average cAUC values for the null model in this second scenario were 0.5.

For modelling the potential distribution of peach palm under current and future climate conditions, we used only records of the domesticated form. For model projections to past climate conditions we used two models (MIROC and CCSM) of the Last Glacial Maximum (LGM) [40] while for future climate conditions, we used 19 downscaled climate models for the periods 2020–2049, 2040–2069 and 2060–2089 and the SRES-A2 emission scenario, obtained from CMIP3 and downscaled by [41]. We restricted the modeled distributions visualized on maps to the maximum training sensitivity plus specificity threshold obtained from model calibration under current climate conditions. To obtain summarizing maps for the different LGM (2) and future (19) climate models we averaged the different threshold-limited suitability maps constructed for all individual climate scenarios. For future projections, we additionally restricted the so-obtained average suitability map to values above the calibration threshold value.

### Mapping genetic and phenotypic diversity

We constructed maps to visualize the spatial distribution of genetic diversity of peach palm in representative sample sites across Central and South America. Neutral molecular data are very useful for the identification of genetic diversity hotspots. Here we considerd the following parameters: allelic richness, observed heterozygocity, and the richness of locally common alleles. Locally common alleles (LCAs) are DNA sequences that occur at relatively high frequency (here >5%) in only a limited portion of the total distribution area of a species (here <25%). An interesting feature of LCA richness is that it can be indicative of the level of historic and/or ongoing genetic isolation of populations, and hence can be informative about the location of potential past refugia [42,43]. Diversity maps were developed with 10 minutes spatial resolution (~18 km at the equator). To allow uncovering diversity gradients in genetic data, we applied circular neighborhoods of two degree diameter around the locations of all the peach palm trees for which genetic data was available [43,44]. In practice this means that each tree was replicated in all the 10 minute grid cells contained in a two degree diameter circle around its location. Since this replication exercise resulted in different numbers of trees per grid cell, in a next step we performed a sample bias correction by calculating cell-based diversity parameters using bootstrapped subsamples (without repetition) of a fixed number of trees per grid cell. Choosing an appropriate sample is subject to a trade-off between calculating genetic parameters based on an as high as possible sample size, which reduces the number of overall grid cells taken into account and hence the spatial coverage, and maximizing the spatial coverage of the diversity maps, which means a greater number of cells but a smaller number of tree samples in each. We tested three minumum sample sizes: 3 trees (the median value of all cells containing trees), 6 and 10 trees per cell. In each scenario all cells with fewer than the minimum number of trees were discarded from the analysis. For each of the retained grid cells, we averaged the values obtained for each of the genetic parameters as calculated for 1,000 bootstrapped samples [43]. As the trends in genetic parameters obtained for the three sample sizes were highly correlated (S3 Table), in what follows we will present the results obtained for a sample size of 3 trees per grid cell in order to maximize spatial coverage.

To increase the geographical coverage of the genetic diversity patterns in peach palm, we performed a similar replication-bootstrapping exercise for allelic richness and locally common alleles based on the molecular marker dataset of Hernández *et al* [37] which included genetic data from four molecular markers (three in common with our study) measured on wild and cultivated individuals classified into 18 geographical populations (20 individuals per population for the cultivated samples, less in the case of the wild types, refer to [37] for further methodological details). After discarding wild samples, we generated maps with the same resolution as described above and the same sample size of 3 trees per cell. To allow for comprehensive analyses of genetic diversity patterns of peach palm, we spatially combined the results from Hernández *et al* [37] with ours. Since both sample sets were genetically characterized in different laboratories and using a different number of markers, we first standardized the cell values of the rasters from each dataset to a common range of 0 to 1 and then merged both raster layers by assigning each cell containing a value from either one or both dataset the highest value among the two.

We applied a similar replication-bootstrapping procedure to our dataset of morphological and biochemical characteristics of peach palm fruits and calculated the average coefficient of variation across all parameters (hereafter referred to as phenotypic variation, for the sake of brevity). The procedure was applied separately on the subset of samples conserved in CATIE and those conserved in INIA, in order to avoid joining samples whose phenotypic variation may have been affected by different environmental conditions at the *ex situ* conservation sites where they are held. Nonetheless, Fisher’s F test and Student’s t-test showed that coefficients of variation calculated on the basis of all individuals in each collection are homoscedastic (F = 0.827; p = 0.6831) and not significantly different between collections (t = 0.313; p = 0.7577), suggesting that the differences in the environment and/or management of the two collections did not significantly affect the extent of the phenotypic variation observed here. To further neutralize possible differences in phenotypic variation between the collections, we standardized the results obtained from the above replication-bootstrapping exercise for each collection separately. We finally merged the rasters of phenotypic variation of the collections with the same procedure described above for the genetic parameters. All calculations and spatial analyses were performed in R [36]; all maps were constructed in ArcMap 10 [45].

### Prioritizing conservation areas

We identified priority areas for on-farm conservation of peach palm based on the geographical intersect between areas for which the ensemble of distribution models returned high suitability scores during present and future (2050s) climate conditions, and areas holding the highest levels of allelic richness. Each of the layers here considered was restricted to the values above the third quartile of their density distributions. Similarly, we considered the geographical intersect between areas for which suitability scores under current and future (2050) climate conditions were high, and areas above the third quantile of the distribution of phenotypic variation (using the dataset resulting from the bootstrapping exercise described earlier), in order to look into priority areas for conservation of peach palm’s morphological and biochemical diversity.

## Results

### Genetic and phenotypic diversity

After removing the three putatively wild samples from our dataset, only 80 of the samples submitted to genetic analyses and 66 of the samples submitted to morphological and biochemical analyses yielded meaningful results. All analysed loci were polymorphic with a total number of 68 alleles; level of polymorphisms per locus ranged from 4 (locus BG-24) to 12 alleles (locus BG-1 and BG-63) with an average of 7.5 alleles per locus. Expected heterozygosity (He) varied between 0.636 (locus BG-17) to 0.881 (locus BG-3). The extent of reduction in observed heterozygosity was used to quantify the level of genetic differentiation between the individuals at each locus, through F statistics (Wright 1951, 1965). The differentiation among individuals at different loci ranged from 0.037 to 0.455 (maximum at locus BG-17) (details on the results of population genetic parameters can be found in S4 Table).

**Fig 3:**
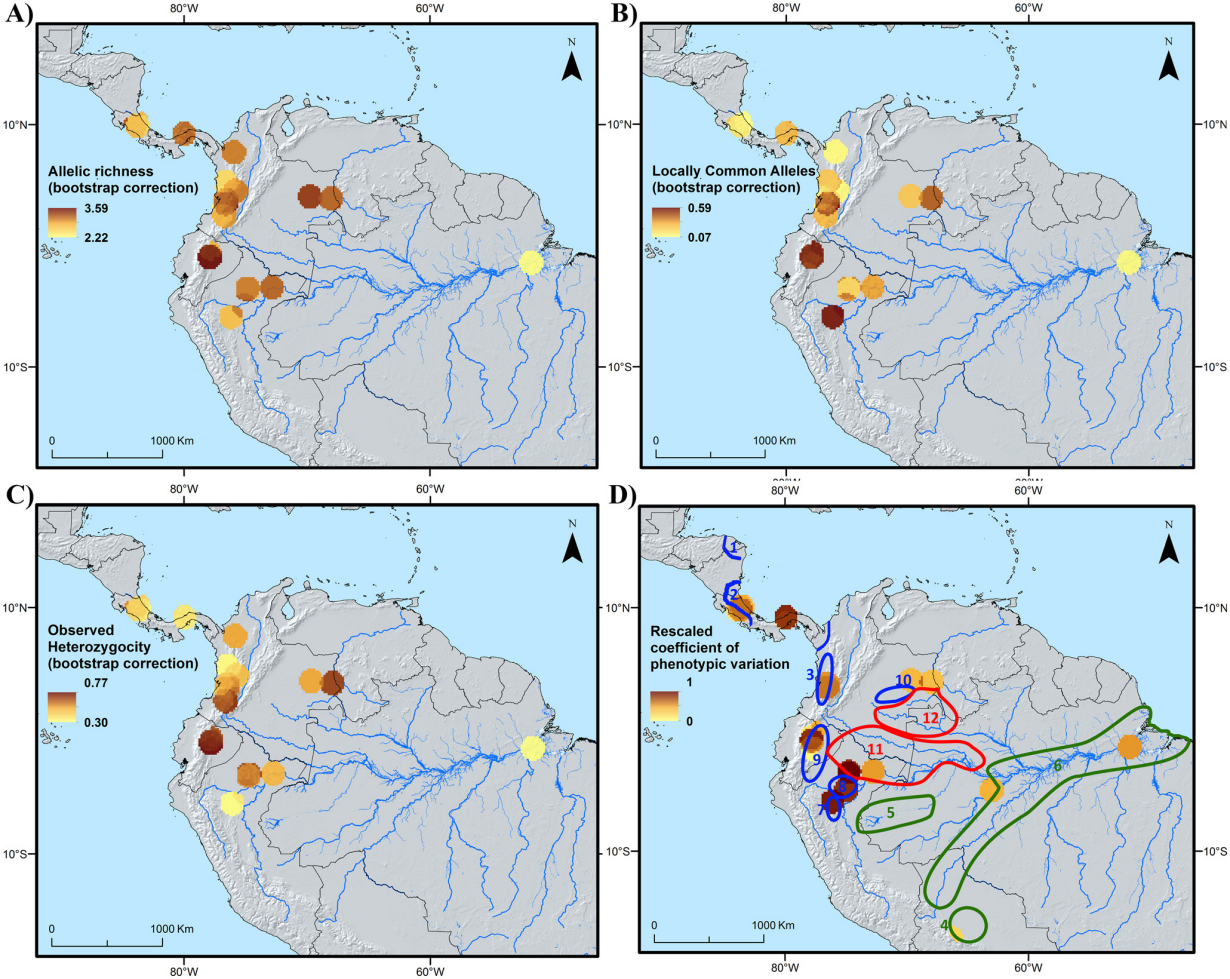
Spatial distribution of allelic richness (A), locally common alleles (B), observed heterozygosity (C) and variation in standardized phenotypic diversity (D), measured as coefficient of variation (st dev/mean) in our *Bactris gasipaes* var. *gasipaes* dataset. The blue, green and red polygons in Fig 3d indicate areas of occurrence of different peach palm landraces [20] (see discussion). Blue polygons enclose the mesocarpa landraces (20–75 gr) Rama (1), Útilis (2), Cauca (3), Pampa Hermosa (7), Tigre (8), Pastaza (9) and Inirida (10); green areas include the microcarpa landraces (< 20 gr) Tembe (4), Juruá (5) and Pará (6); and red polygons refer to the macrocarpa landraces (75–200 gr) Putumayo (including Solimões, 11) and Vaupés (12). It is important to note that the many locations for which only one accession was included in the phenotypical characterization are not included in the Fig 3d because the coefficient of variance can only be calculated for two or more individuals.

**Fig 4:**
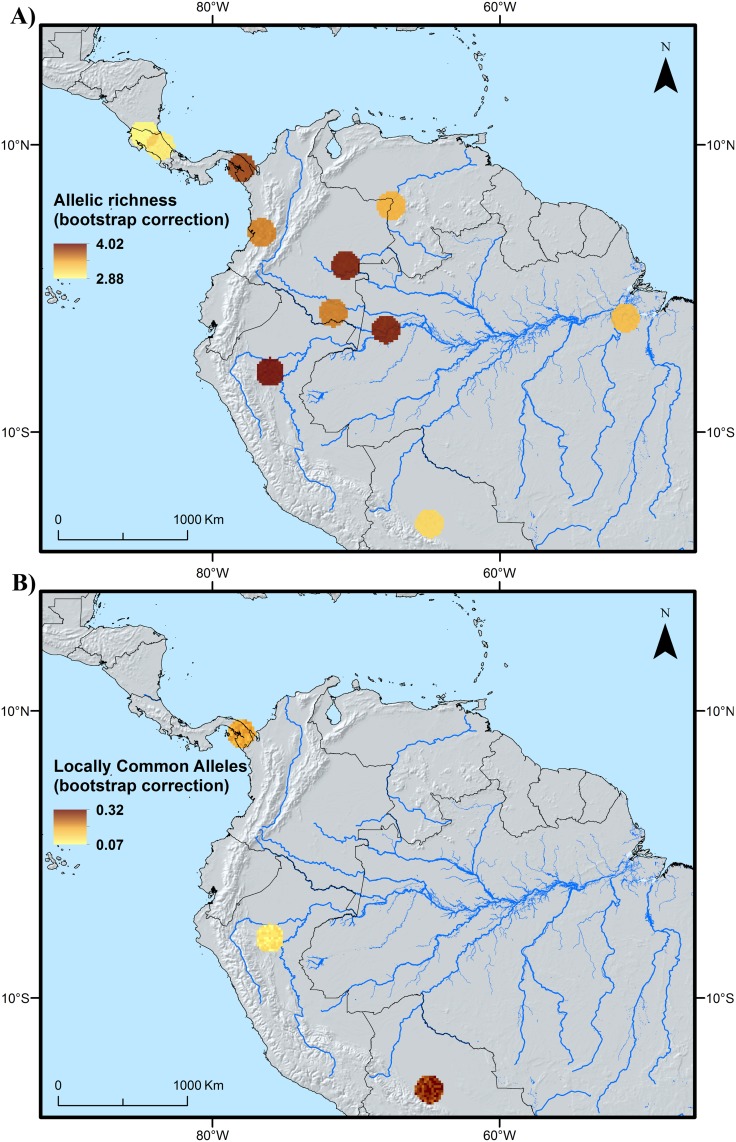
Spatial distribution of allelic richness (A) and locally common alleles (B) based on the cultivated samples (~20 individuals per population) from the *Bactris gasipaes* var. *gasipaes* dataset of Hernández *et al* [37].

Distribution patterns of genetic diversity show that both allelic richness and observed heterozygosity reach their highest values in the area covering the Ecuadorian Amazon (Fig 3A and 3C, respectively). Lower but still notable allelic richness is observed among the samples from the Colombian Llanos (Vichada department; Fig 3A). A map of allelic richness based on the dataset from Hernández *et al* [37] shows high richness in the Peruvian and western Brazilian Amazon. This data also highlights high allelic richness in Panama, close to the border with Colombia (Fig 4A). Highest richness of locally common alleles according to the present study’s dataset are found in the Ecuadorian and northern Peruvian Amazon, followed by the Cauca area and the Llanos in Colombia (Fig 3B), while the dataset from Hernández *et al* [37] detects a hotspot in central Bolivia (Fig 4B). The fact that the values obtained for LCA and allelic richness did not always perfectly align at all sampling sites, both within our data and between our dataset and that of Hernández *et al* [37], is likely due to the fact that the different parameters focus on different aspects of genetic diversity, and possibly also to the small sample size we used, which exposes measures to a certain degree of stochasticity.

The areas holding highest fruit morphological and biochemical diversity among those the present study covers are found in the northern Peruvian and Ecuadorian Amazon followed by southern Panama (Fig 3D). Additional maps displaying the spatial distribution of the variation of phenotypic traits for the CATIE and INIA collections separately, as well as of single morphological and biochemical traits for each collection are shown in S1 to S3 Figs.

### Past, present and future habitat suitability

**Fig 5:**
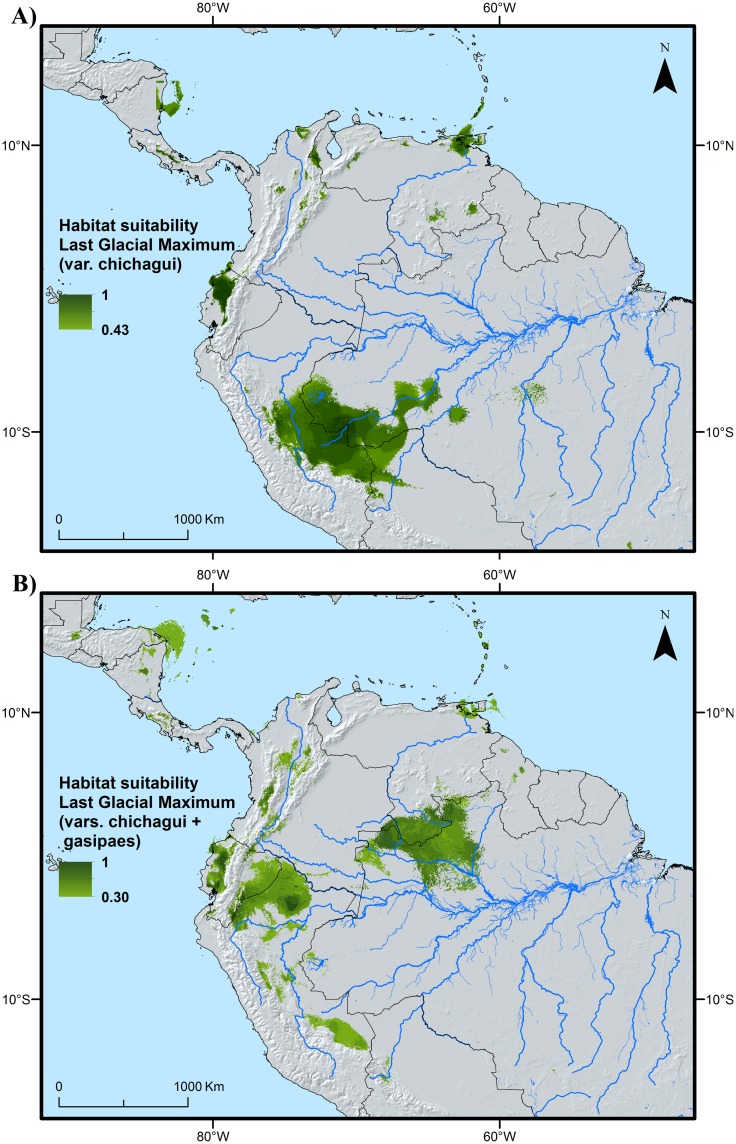
Putative distribution of suitable habitat of peach palm during the Last Glacial Maximum. A: results of model calibration undertaken based on the wild form only (*Bactris gasipaes* var. *chichagui)*; B: results of calibration based on both wild and cultivated (*Bactris gasipaes* var. *gasipaes*) trees pertaining to the ecological niche occupied by var. *chichagui*.

Different LGM suitability maps were obtained from model calibrations based on wild type points only, versus a combination of wild and cultivated (Table 1). Under the first scenario, suitable habitat conditions were detected in the Ecuadorian Pacific coast and southwestern Amazonia, as well as in some scattered areas in the northern part of south America (Fig 5A). Under the second scenario, the distribution of suitable habitat stretches out over fragmented areas across coastal Ecuador, western Amazonia up to western Colombia, and southern Venezuela—northern Brazil (Fig 5B).

**Table 1:** Metrics of model calibrations and evaluation under current environmental conditions for projections to past and future climate conditions.

	Model calibration for projection to past		Model calibration for projection to future
	Only wild (var. *chichagui*) records (55)	Wild (var. *chichagui*) and cultivated (var. *gasipaes*) records (55+111)	Only cultivated (var. *gasipaes*) records (166)
Model ensemble	MAXENT	MAXENT & GBM & RF & RPART	GBM & MAXENT & GBMSTEP & FDA & EARTH & GAM & RF & GLM & MGCV & GLMSTEP & BIOCLIM
AUC	0.84	0.99	0.97
Calibrated AUC	0.60	0.59	0.87
Maximum training sensitivity plus specificity threshold	0.43	0.30	0.10

**Fig 6:**
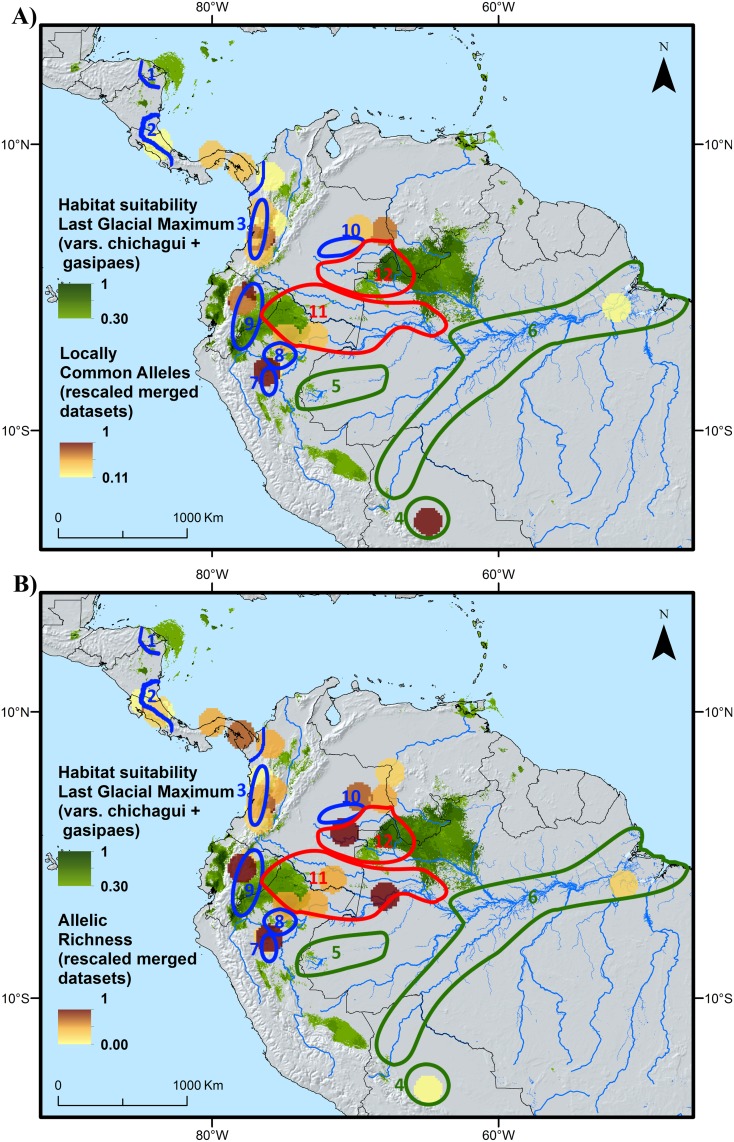
Distribution of potential LGM refugia of peach palm (green areas) and distribution of rescaled locally common alleles (A) and rescaled allelic richness (B). The genetic data visualized is based on a spatial combination of the results from the present study and that of Hernández *et al*[37]. The blue, green and red polygons indicate areas of occurrence of different peach palm landraces (see discussion) [20]. Blue polygons enclose the mesocarpa landraces (20–75 gr) Rama (1), Útilis (2), Cauca (3), Pampa Hermosa (7), Tigre (8), Pastaza (9) and Inirida (10); green areas include the microcarpa landraces (< 20 gr) Tembe (4), Juruá (5) and Pará (6); and red polygons refer to the macrocarpa landraces (75–200 gr) Putumayo (including Solimões, 11) and Vaupés (12). Several LGM suitable areas are not visible as they graphically coincide with the extent of the circular neighbourboods of the genetic data. This is the case in particular for the circular neighborhoods overlapping with the polygons describing the distribution of the Pampa Hermosa and Tigre landraces.

Based on the data available to us, we believe that the second scenario (Fig 5B) is most useful to help understanding the distribution of intraspecific diversity in peach palm. The combination of this LGM distribution scenario with the genetic characterization data and the distribution of known landraces in peach palm [11] reveals two fairly consistent spatial patterns (Fig 6A and 6B). First, each of the areas where the highest values of genetic diversity parameters (LCA and/or allelic richness) were observed tends to be associated with the distribution of one particular traditional landrace [20,21]. This is the case for the hotspots in central Bolivia (Tembe landrace), northern Peru (Tigre and/or Pampa Hermosa), Ecuador (Pastaza), northwestern Brazil (Solimões-Putumayo), eastern Colombia (Inirida and/or Vaupés), western Colombia (Cauca) and Panama (Útilis). Second, most of these genetic diversity hotspots either overlap or are adjacent to areas where habitat conditions are likely to have remained suitable for peach palm’s wild ancestor during the LGM. Two interesting cases exist to further test the validity of these two spatial patterns. First, there is the area from Pucalpa, Peru, to the northwestern Acre state of Brazil, which is home to the Juruá landrace and where our LGM model also predicted suitable habitat conditions. Second, the area in the Peruvian Cuzco and Madre de Dios departments highlighted in Fig 6 as holding suitable habitat during the LGM is spatially close to the southwesternmost distribution of the Pará landrace. Based on the patterns observed in similar sites across the western Amazon one would expect to find high levels of allelic richness or locally commom alleles, or both, in both these areas. Genetic characterization of peach palm from these areas are necessary to confirm or refute this.

**Fig 7:**
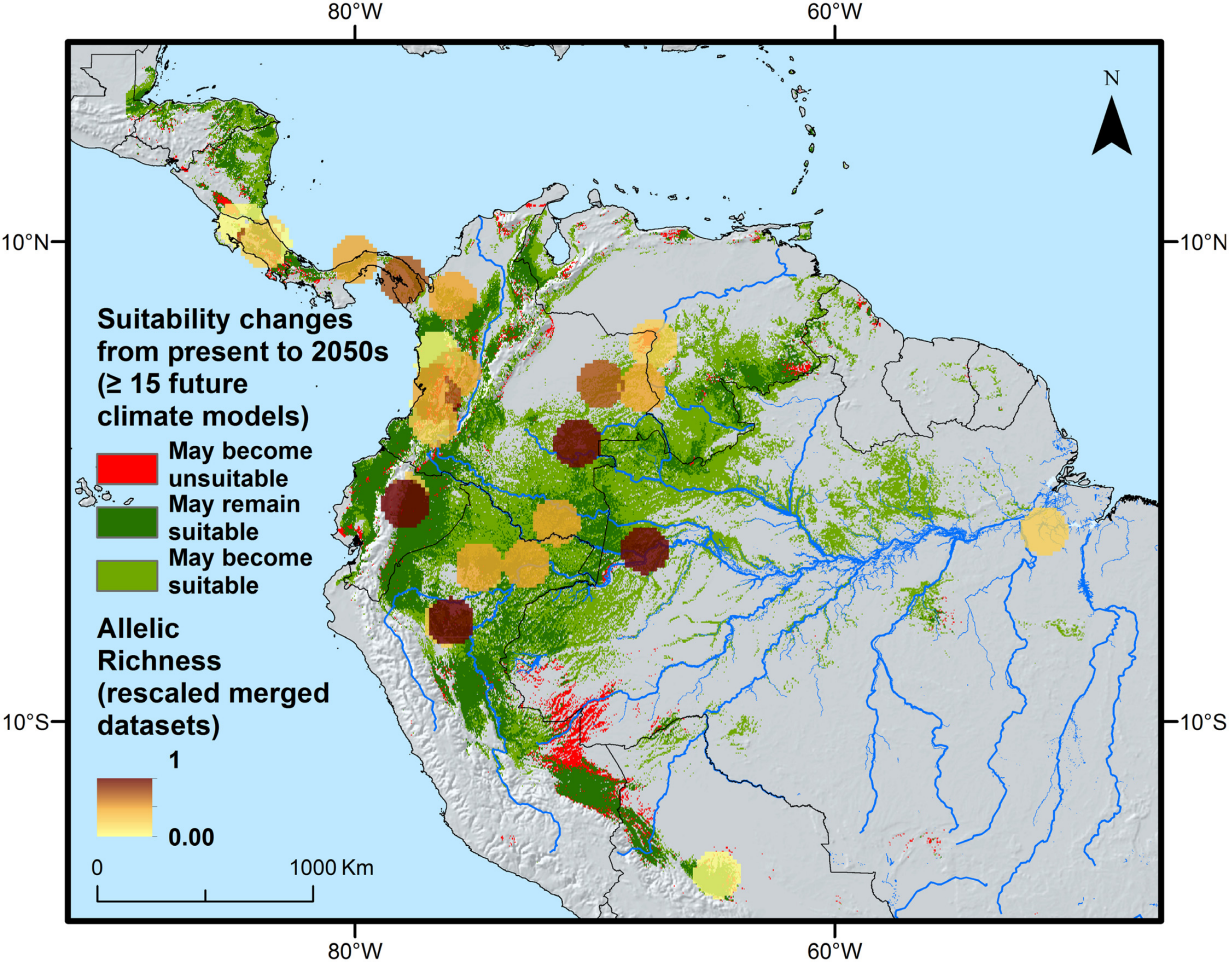
Expected changes in habitat suitability for cultivated *Bactris gasipaes* var. *gasipaes* in the future compared with hotspots of allelic richness based on a combination of our genetic dataset with that of Hernández *et al* [37]. The suitable areas shown here largely correspond to the overall area for which the modelling ensemble predicts habitat suitability for at least 15 out of the 19 different future climate models considered.

The expected changes in habitat suitability from present to the 2050s allow assessing the potential impact of climate change on the distribution of suitable habitat for peach palm. It appears that climate change may lead to a net increase in suitable habitat for peach palm, with very few areas predicted to lose their current suitability (Fig 7). Also, the main hotspots of allelic richness are not expected to be heavily affected by climate change, providing positive perspectives for the on-farm conservation of peach palm.

**Fig 8:**
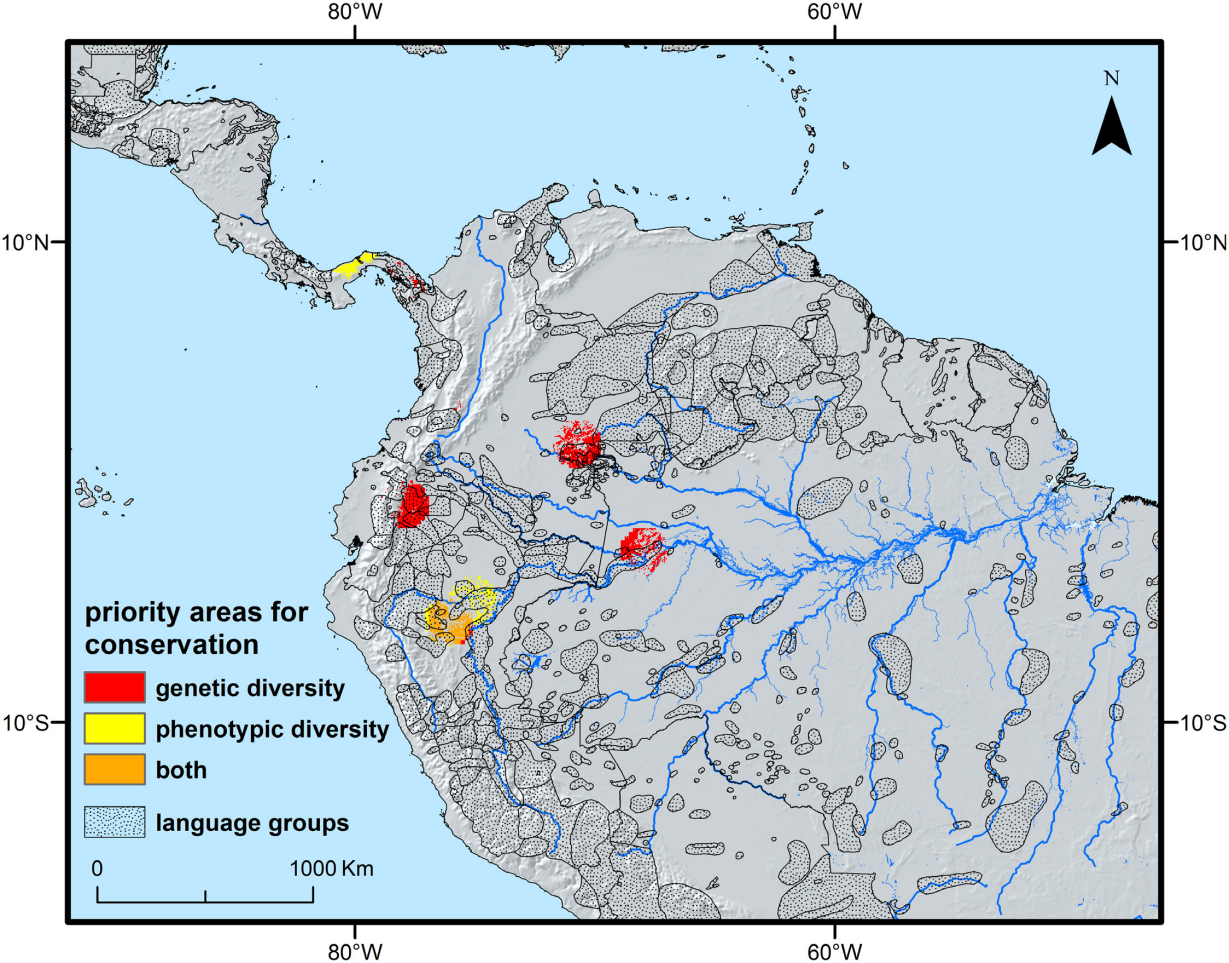
Priorities for on-farm conservation of areas rich in genetic and phenotypic diversity and likely to remain suitable so in the future (in red and yellow, respectively). **Dotted** polygons represent areas likely to be occupied by indigenous peoples (based on language maps from the Ethnologue database [47]), suggesting sites where on-farm conservation may be already carried out by local communities or could be strengthened through their involvement.

Fig 8 shows the location of priority areas for on-farm conservation of peach palm genetic resources, based on the combination of the genetic and phenotypic diversity data and climate suitability modeling. The area around Tarapoto in the northern Peruvian Amazon is priority for the conservation of both genetic and phenotypic diversity of peach palm; Amazonian Ecuador, the Vichada region in Colombia, the Solimões in region in Brazil and eastern Panama are more strategic for the conservation of genetic diversity, while the Pacaya-Samiria reserve in the northern Peruvian Amazon and Panama is more stategic for phenotypic diversity. Fig 8 shows that the south American priority areas overlap with the distribution of present-day language groups, which we considered a proxy for the presence of indigenous peoples. Any on-farm conservation strategy depends on people, whose management practices influence the genetic and phenotypic diversity dynamics of the plant species they use [46]. When developing on-farm conservation plans for the priority areas identified in Fig 8, it would be worth seeking the participation of, among others, the Quichua, Huaorani and Achuar groups whose territories overlap with the priority areas in Amazonian Ecuador, the Cubeo, Curripaco and Yuriti people in the Colombian Vaupés department, the Chayahuita, Quechua, Urarina, Cocama-Cocamilla and Shipibo-Conibo for the priority areas in the northern Peruvian Amazon, the Ticuna and Mirana people for the areas identified in western Brazil, and the Kuna and Embera people from Panama.

## Discussion and Conclusions

### The distribution of peach palm genetic diversity

Our results suggest an overall tendency to higher levels of genetic diversity in South American, and particularly western Amazonia, compared to Central American samples, corroborating the hypothesis of South America as being the region of the species’ earliest domestication [7,8,11,15,20,21,48]. Interestingly, a fair degree of congruence exists between our present findings for peach palm and those reported for cacao (*Theobroma cacao* L.) [43]. While no data were available for the latter species from Colombia or Bolivia, the highest levels of genetic diversity of cacao were observed in Amazonian Ecuador, the area around Tarapoto in the Peruvian Amazon, and the Solimões region in northwestern Brazil, in a similar fashion as reported here for *Bactris*. Another area of congruence is the northern Peruvian Amazon, where the highest levels of phenotypic diversity of our peach palm dataset was observed, confirming earlier observations [17]. In this very same area the highest diversity of genetic clusters in cacao (which to some level are comparable to landraces in peach palm; see [43]) were found, which the authors related to the fact that the northern Peruvian Amazon region was an important center of crop genetic resources at the time of European conquest [20,49]. Such centers are known to have concentrated and created crop diversity through trade, cultivation and selection to guarantee their inhabitants’ subsistence and survival. In peach palm, the legacy of such human-mediated processes might be manifested by the convergence of many different landraces in this region (Fig 2), such as the Pampa Hermosa, Yurimaguas, Tigre and Putumayo [21].

On the other hand, the combination of high genetic and phenotypic diversity in these landraces might additionally relate to longstanding indigenous management practices as suggested by ethnobiological studies in the Huaorani indigenous group from the Ecuadoran Amazon, another hotspot of both genetic and phenotypic peach palm diversity (Fig 3A and 3D). The Huaorani’s reverence to peach palm [50] led them to maintain sacred groves in which they established new plants from seed, thus promoting geneflow into and among ancestral groves over time, particularly within the local Pastaza landrace, but constraining it to occur only within specific sub-populations established by groups with shared kinship (i.e. one group’s ancestral groves does not contribute alleles to an unrelated group’s groves) [51]. In the long-term such management practices likely result in an increase of phenotypic diversity across groves, while at the same time maintaining high levels of genetic diversity within the overall regional metapopulation.

The apparent compatibility of diversity patterns in peach palm and cacao is likely due the fact that they have similar habitat requirements and their distributions may have been influenced by early humans in similar ways. Both species are very characteristic elements of homegardens in Amazonia [52,53]. However, an important difference is that in peach palm a clear distinction exists between domesticated (var. *gasipaes*) and wild (var. *chichagui*) populations, while in cacao such a distinction is much less straightforward [43]. This has implications for the identification of potential drivers underlying contemporary patterns in the diversity distribution of peach palm, whose natural and human history is likely more complex.

### The domestication of peach palm: a hypothesis

Drawing on the present results, and building on previous findings reported in literature, in what follows we present a hypothesis of how peach palm may have evolved from a wild species into the cultivated landraces known today across Latin America. While acknowledging that our dataset is too small to unmistakingly validate this hypothesis, we hope that it can inspire future research aimed at furthering our understanding of the temporal and spatial dynamic of this important Amazonian crop.

Domesticated var. *gasipaes* clearly did not exist at the time of the last glacial period; therefore, explaining the congruence between genetic diversity patterns in the domesticated variety and LGM refugia requires a closer look at peach palm’s wild types, particularly types 1 and 3 which are considered to have been involved in the domestication process [8–11,20]. Hernández Ugalde *et al* suggested that the distribution patterns of wild peach palm were likely to have been strongly influenced not only by the last glacial period, but also by geological events during the Pliocene and Miocene, possibly leading to genetic differentiation of isolated palm populations [8]. While this may have led to the genetic diferentation between wild types 1 and 2 we believe it is unlikely that type 3 already existed before the arrival of the first human populations.

Human occupation of South America may have started as early as 22,000 BP [54] and human influence on the Amazon forests dates back at least 13,000 years [55] Soon after the end of the last Pleistocene glacial period, human settlement of the Neotropics began to change from more sparsely distributed and short-term occupations to more “settled” landscapes, which ancient people manipulated and altered by creating clearings in forests through the use of fire [56,57]. Food production began in a number of localities in tropical Central and South America during the early Holocene (between 9,000 and 5,600BP), not long after the climate and vegetation underwent profound changes with the ending of the glaciation [58]. More intense human modification of the landscape and its plants is likely to have kick-started the domestication of peach palm early on, possibly through an initial focus on the species’wood qualities [51]. While our results do not allow identifying the location of peach palm’s initial domestication, it is likely that it must must have taken place in some area of the broad region in Western and South Western Amazonia where wild types 1 and 3 overlap. Considering that until insufficient evidence for multiple initial domestication events is available, a single origin hypothesis is to be preferred as the most parsimonious [20,59]. Most previous studies have argued in favour of the hypothesis that the domestication of peach palm most likely started in a single area in southwestern Amazonia [8–10,18–21], which was recently confirmed by a study that used chloroplast maker data of both cultivated and wild samples [9]. We therefore embrace the likelihood of a southwestern Amazon orgin, although our hypothesis does not exclude the possibility of an initial domestication event in other areas of western Amazonia.

Regardless of the actual location were it took place, it is likely that this initial domestication event resulted in the emergence of the wild type 3, as an incipient domesticate from a type 1 progenitor [60], which was then further selected through secondary domestication processes, leading to the landraces known today. Incipient domesticates are those populations which have undergone a human-influenced founder event that reduces their genetic diversity, while their phenotypic diversity varies only somewhat from the ancestral wild population in the traits selected by humans [20]. In peach palm, such a founder effect may have taken place when early humans selected a small sample of the genetic diversity available in a source population and moved it into an area that lacked natural peach palm populations; here, the bottlenecked genepool would have been further subjected to human selection pressures targeting larger fruit sizes, resulting in type 3 phenotypes. Assuming a southwestern initial domestication event, the source populations are likely to have been one or more of those associated with the putative glacial refugium in the Cuzco-Madre de Dios area (Fig 6), but a similar reasoning can be applied to other putative refugia in western Amazonia.

Cristo-Araújo *et al* [9] advocated that after initial domestication in southwestern Amazonia, peach palm was later dispersed across two main routes, one across western Amazonia into Central America, and one across the Madeira river to central and eastern Amazonia. While we generally concur with this argument, there must have been a considerable time lag between both routes, the first one involving the dispersal of type 3 which occurred ahead (early Holocene) of the second route along which the by then already domesticated var. *gasipaes* was spread (late Holocene). Clement *et al* [51] similarly argued that dispersal along the eastern route was later and slower.

Our model assumes that the incipiently domesticated type 3 formed the basis of secondary domestication processes leading to *B*. *gasipaes* var. *gasipaes* through continued human selection and hybridization with (progenitor) type 1, a hypothesis on which many authors share consensus [10,11,17,61,62]. We believe that these secondary domestication processes were spread out over different geographical areas across western Amazonia to which type 3 germplasm was introduced by humans early on (from southwestern Amazonia, or any other potential area in western Amazonia) and where populations of type 1 likely ocurred naturally. Indeed, if our past distribution model is correct, type 1 could have survived in multiple refugia across the western Amazon, which would probably have led to genetic differentiation between isolated populations, in a similar fashion as for cacao and Brazil nut [43,63]. Introgression of genes from local type 1 populations (each carrying a distinct genetic makeup) in introduced type 3 trees, in combination with different human selection processes in areas where type 1 populations strived, would have resulted in the diverse landraces known today [38,64]. The fact that nearly all the hotspots of genetic diversity we have identified in western Amazonia (i) overlap or are adjacent to areas where peach palm’s progenitor (type 1) may have survived and genetically differentiated during the last glacial period, and (ii) are associated with one or two landraces (Fig 6A and 6B), corroborates this hypothesis.

Type 1 still occurs in vast areas across western and southern Amazonia, covering most of the areas in western Amazonia where we have observed hotspots of genetic diversity. It is possible that it used to occur somewhat further to the north (northern Brazil) and west (Amazonian Ecuador and northern Peru) at the time secondary domestication processes were initiated in these places. There is even the possibility that it still occurs in these areas but that botanical collections have missed it until now. For example, the Brazilian RADAM collection contains a potential var. *chichagui* observation as far north as the Japurá River (R08; [6]) which is geographically very close to the putative refugium located in northwestern Brazil and southern Venezuela (Fig 6). Similarly Mora-Urpí [17] described a wild type “Capu” at the Adean foothills of Amazonian Ecuador, in the area where the Pastaza landrace is currently found (Fig 6). The fact that in the western distribution area of the Putumayo landrace genetic diversity levels were generally low might either indicate that type 1 did not occur there in spite of LGM suitability, or that the Putumayo landrace was domesticated in the eastern part of its distribution and was then spread westward.

Several studies have observed the ease of introgression between natural and introduced cultivated peach palm [38], resulting in greater similarity between cultivated and nearby natural populations than between geographically more distant cultivated populations [8,21,38]. Similar observations exist for other species such as the genus *Leucaena* in which human-mediated sympatry in Mesoamerican “backyard gardens” of previously separated wild taxa, prolonged predomestication cultivation followed by spontaneous hybridization led to the emergence of different domesticated species [65]. According to this hybridization hypothesis, as local societies continued the domestication process and created the distinct landraces of var. *gasipaes* across western Amazonia, genes from the original wild populations that persisted and genetically differentiated in local refugia were incorporated in the landrace populations, leading to increased levels of allelic richness (Fig 6A) and in some cases of LCAs (Fig 6B).

If our assumption of early human-mediated movement of type 3 across Western dispersal routes is correct, present-day occurrence of type 3 on both sides of the Andes (including the Colombian Cauca area) may similarly be explained by human dispersal across the mountains [1], in a similar fashion as for cacao [66]. There is evidence of contact between Amazonian and coastal Ecuador since at least 5,000 years, testified by ceramics and marine shells found on the Amazonian side of the Andes [67]. The finding of Hernandez-Ugalde *et al* [37] that wild peach palm from coastal Ecuador (type 3) is genetically similar to the Cauca landrace as well as to central American landraces corroborates this hypothesis. Movement of the incipiently domesticated type 3 across the Andes and into Central America likely involved more or less serious genetic bottlenecks, possibly explaining the lower levels of diversity detected in this region (Fig 6). One explanation for the slightly elevated allelic richness in Southern Panamá detected by Hernández *et al* [37] may be due to more recent introduction of diverse materials from different origins to the area. Movement of peach palm material in more advanced stages of domestication than type 3 is also likely to have taken place on both sides of the Andes, as a result of pre-Columbian trade and migration [51,68].

In southwestern Amazonia, hybridization between one or more populations of incipiently domesticated type 3 trees and type1 populations that may have survived in the putative LGM refugium in the Cuzco-Madre de Dios region may have given rise to the Pará landrace. This is supported by the finding that wild peach palm samples (likely type 3) from Acre, Brazil (geographically very close to the abovementioned refugium) were genetically very similar to samples from the Pará landrace [37,69]. A similar argument may apply for the Tembe landrace which is genetically similar to the Pará landrace and the Acre wild samples, possibly through hybridization between type 3 and type 1 populations that may have persisted in one or more refugia in eastern Bolivia. The relatively late dispersal of the Pará landrace along the Madeira River into Central and Eastern Amazonia [51] (as opposed to type 3 dispersal across western Amazonia) may coincide with the emergence of sedentary lifestyles in Amazonia some 4,000–3000 years ago, when plant cultivation became the major source of subsistence and allowed agricultural societies to colonize new areas. Thomas *et al* [63] have similarly argued that human dispersal of Brazil nut (*Bertholletia excelsa*), another Amazonian species important for non-timber forest products, from southwestern source populations may have coincided with the expansion of horticulturalist societies towards the northeast. The fact that the distributions of both peach palm and Brazil nut in central and eastern Amazonia are concentrated along the main rivers and their tributaries [51,63] and show some association with the occurrence of Amazonian Dark earth soils [55,63], which were created 3,000–500 years ago [70] supports this hypothesis.

One of the the best ways to test our tentative domestication model would be to genetically characterize naturally occurring type 1 and type 3 populations across their ranges (on both sides of the Andes), and most importantly in each of the convergence areas where (i) high diversity levels have been observed in domesticated peach palm and (ii) suitable habitat conditions prevailed during the LGM. This would then allow for a more systematic comparison of the genetic makeup of local wild and domesticated populations, and a better evaluation of the putative association between genetically differentiated populations of type 1 and potential LGM refugia. The preferential use of chloroplast DNA markers in particular would be beneficial since nuclear markers are biparentally inherited and hence are subject to recombination. On the contrary, chloroplast DNA is almost always maternally inherited, which avoids the issue of recombination and is more appropriate for identifying the location of the domestication event(s) that gave rise to peach palm’s landraces.

### On-farm conservation

Suitability modeling of peach palm under different climate change scenarios suggests a positive future. This finding is in line with a recent continent-wide assessment of the relation between species range sizes and their resilience to climate change in the Americas. The authors found that species with wide distributions, such as peach palm, are generally habitat generalists and therefore better armed to deal with a changing climate [71]. Similar trends were recently found for Brazil nut [72]. The optimistic forecasts suggest that on-farm conservation could be an effective conservation strategy for peach palm. This is particularly true in light of the high costs involved in establishing and maintaining *ex situ* field collections [73–75] although these can be mitigated by the establishment of core-collections representative of the overall diversity and by giving this core priority in maintenance [76]. While the priority areas identified here only apply to the samples we have analysed, the challenge remains to carry out a similar exercise taking into account more genebank accessions and other collections that have been genotyped in the past. As done here, multiple criteria should be taken into account, such as the probability of climate stability in the future, the occurrence of high levels of intraspecific diversity, as well as the presence of indigenous and local communities with whom participatory conservation schemes should be developed, implemented and monitored. In addition, a careful analysis of threats other than climate change, particularly anthropogenic disturbances [77] such as fire and expansion of agricultural land [6], that may put at risk the on-farm conservation and viability of priority populations, should be undertaken.

In conclusion, our preliminary results highlight the usefulness of spatial analyses for making molecular data more explicit and meaningful within the broader context of geography, landscapes, climate dynamics, and human history and culture. Further validation of the initial hypotheses presented here for peach palm is needed, requiring the assemblage of larger genetic and phenotypic datasets at continental level (of both wild and cultivated populations), the use of standardized molecular markers (both nuclear and chloroplast) and protocols across different datasets and regions to facilitate aggregate analyses.

## Supporting Information

S1 Fig Distribution of variation in phenotypic traits measured for each collection separately. (TIF)

S2 Fig Distribution of individual phenotypic traits measured for each collection separately. (TIF)

S3 Fig Distribution of individual phenotypic traits measured for each collection separately (continued). (TIF)

S1 Table Plant material from the three sources (CATIE, INIA and Colombian on farm collection). (DOCX)

S2 Table List of primer sequences for the microsatellite loci analysed, the expected product and the number of alleles. (DOCX)

S3 Table Results of correlation tests between different sample sizes (numbers of trees per cell) for allelic richness and locally common alleles measured on the dataset of *Bactris gasipaes* var. *gasipaes*. (DOCX)

S4 Table Summary of population genetic parameters. Allele number, average allele frequency, observed and expected heterozygosity, and the fixation index Fis were measured on *Bactris gasipaes* var. *gasipaes* accessions from CATIE, INIA and those collected on-farm in Colombia.
(DOCX)

S1 Text Genetic data collection. (DOCX)

S2 Text Phenotypic data. (DOCX)

S3 Text Suitability modeling. Models considered and calibration methods.
(DOCX)
