# Supplementary figures and images for: An Integrated Hypothesis on the Domestication of Bactris gasipaes
Source: PLoS One. 2015 Dec 10;10(12):e0144644. doi: 10.1371/journal.pone.0144644 (PMC4675520; doi:10.1371/journal.pone.0144644)

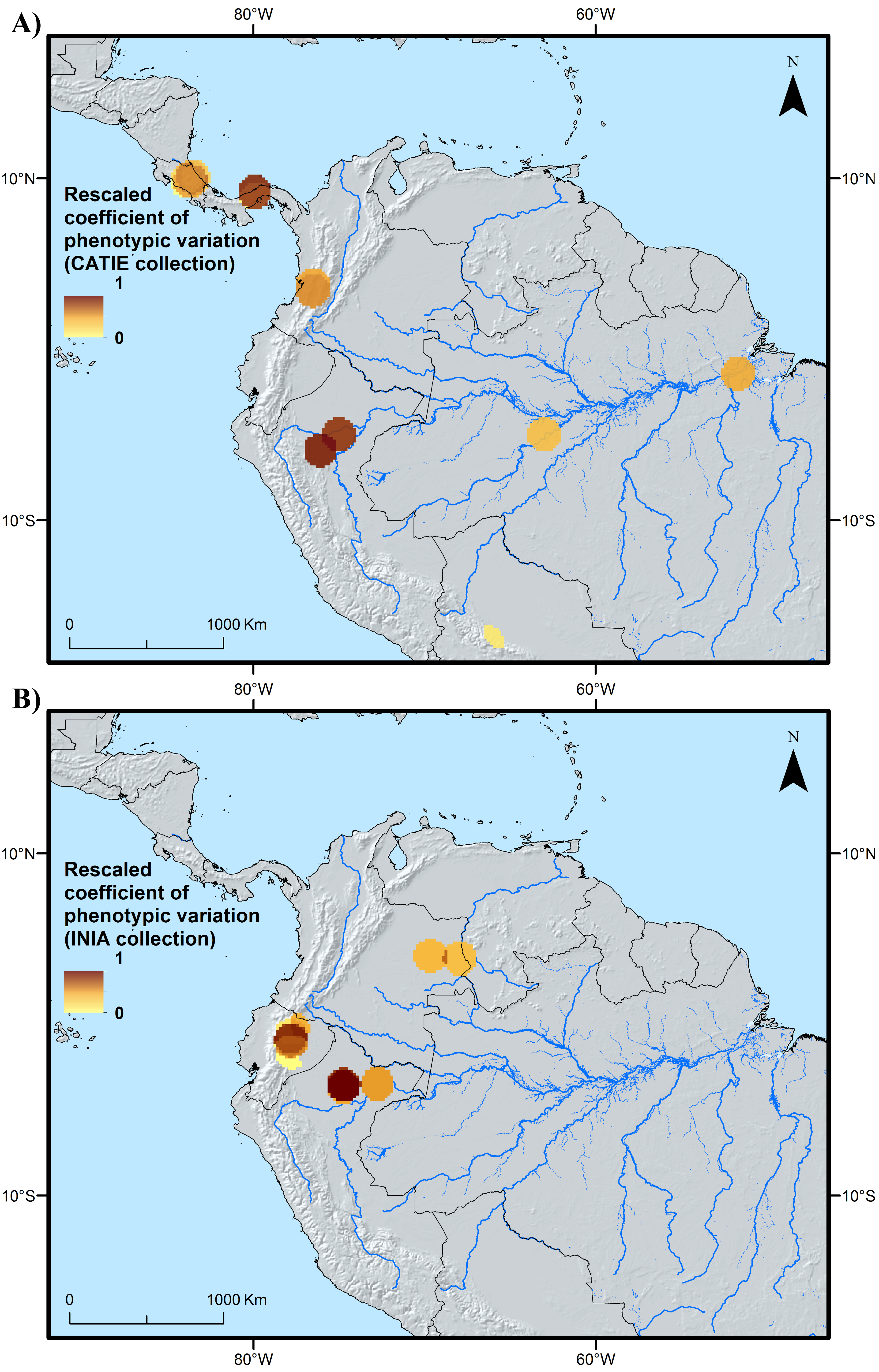

Supplement: S1 Fig — (TIF) [file pone.0144644.s001.tif]

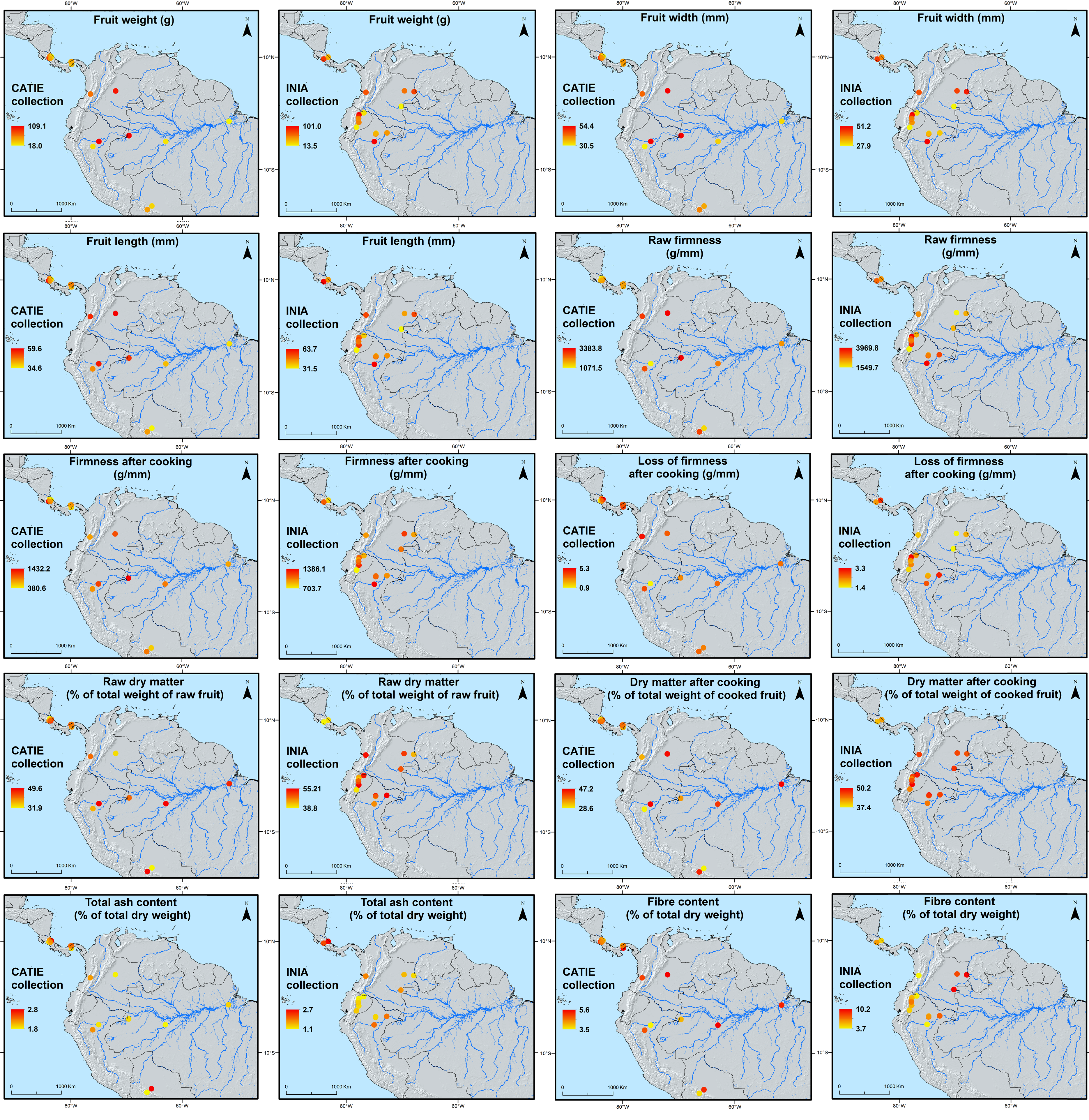

Supplement: S2 Fig — (TIF) [file pone.0144644.s002.tif]

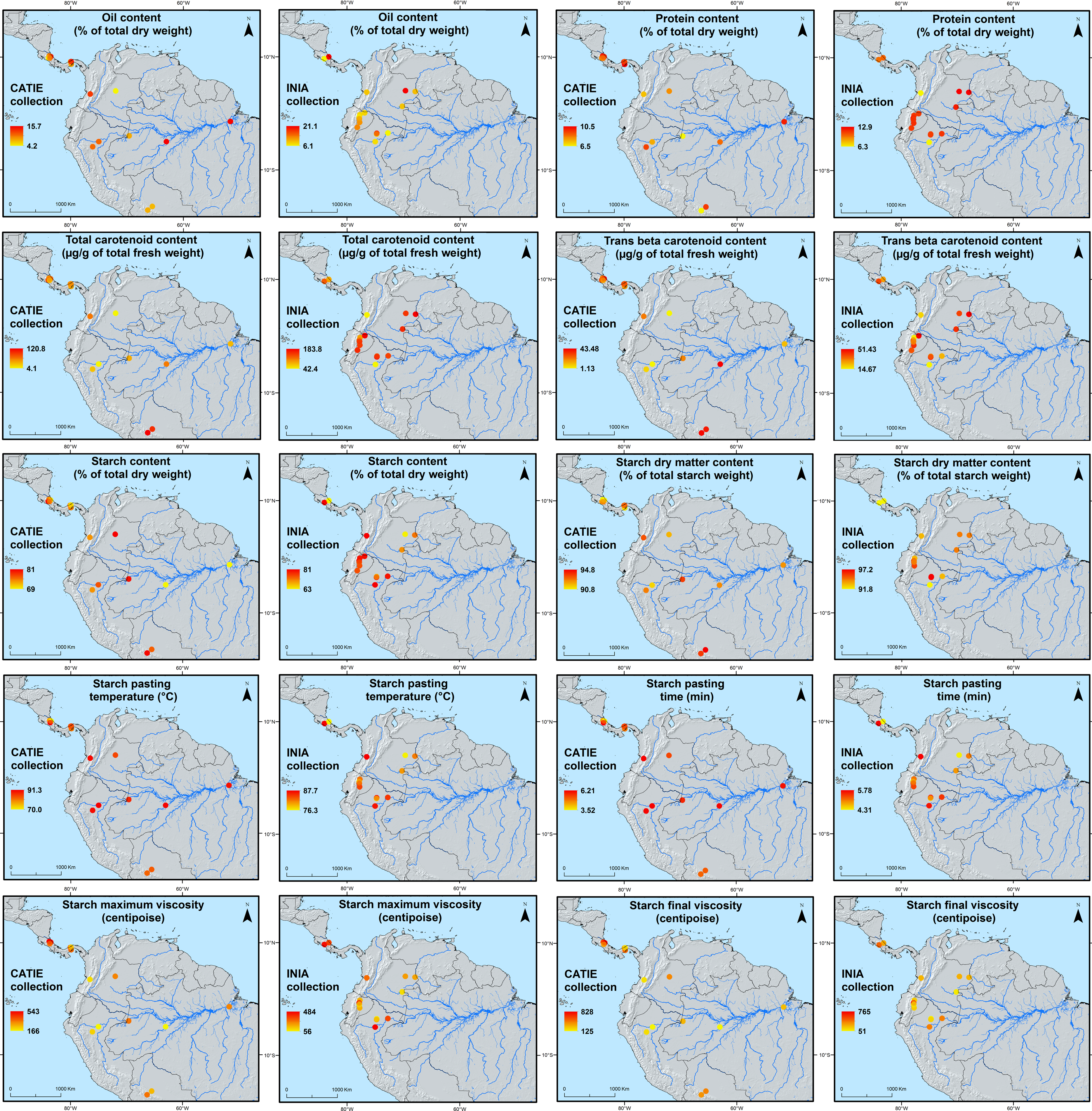

Supplement: S3 Fig — (TIF) [file pone.0144644.s003.tif]
