# Supplementary material for: An Integrated Hypothesis on the Domestication of Bactris gasipaes
Source: PLoS One. 2015 Dec 10;10(12):e0144644. doi: 10.1371/journal.pone.0144644 (PMC4675520; doi:10.1371/journal.pone.0144644)
Supplement: S1 Table — (DOCX) [file pone.0144644.s004.docx]

| **Accession** | **Country of origin** | **Administrative level 1** | **Administrative level 2** | **Locality** | **Lat** | **Long** | **Source** |
| --- | --- | --- | --- | --- | --- | --- | --- |
|  |  |  |  |  |  |  |  |
| 12806-2 | Bolivia | Cochabamba | Chapare | - | -16.5000 | -65.5000 | CATIE |
| 12807-1 | Bolivia | Cochabamba | Chapare | - | -17.1500 | -66.3166 | CATIE |
| 12804-2 | Brasil | Alto Solimões | Tabatinga | Aldeia Umariacu De Tabatinga | -3.9628 | -69.6025 | CATIE |
| 12509-2 | Brasil | Amazonas | Amaturá | - | -5.0000 | -63.0000 | CATIE |
| 14461-1 | Brasil | Para | - | Gurupá | -1.4166 | -51.6500 | CATIE |
| 14454-1 | Brazil | Para | - | - | -1.4166 | -51.6500 | CATIE |
| 14457-1* | Brazil | Para | - | - | -1.4166 | -51.6500 | CATIE |
| 12-EL TAMBO | Colombia | Cauca | El Tambo | - | 2.5000 | -77.0000 | on-farm Colombia |
| 3-POPAYAN | Colombia | Cauca | Popayan | - | 2.4381 | -76.6131 | on-farm Colombia |
| 6-POPAYAN | Colombia | Cauca | Popayan | - | 2.4381 | -76.6131 | on-farm Colombia |
| 11-POPAYAN | Colombia | Cauca | Popayan | - | 2.4381 | -76.6131 | on-farm Colombia |
| 13-POPAYAN | Colombia | Cauca | Popayan | - | 2.4381 | -76.6131 | on-farm Colombia |
| 21-POPAYAN | Colombia | Cauca | Popayan | - | 2.4381 | -76.6131 | on-farm Colombia |
| 7-MUMBU | Colombia | Choco | Mumbu | - | 5.3000 | -76.3833 | on-farm Colombia |
| 9-MUMBU | Colombia | Choco | Mumbu | - | 5.3000 | -76.3833 | on-farm Colombia |
| 2-RIO SUCIO | Colombia | Choco | Rio Sucio | - | 7.6747 | -75.8656 | on-farm Colombia |
| 4-RIO SUCIO | Colombia | Choco | Rio Sucio | - | 7.6747 | -75.8656 | on-farm Colombia |
| 8-RIO SUCIO | Colombia | Choco | Rio Sucio | - | 7.6747 | -75.8656 | on-farm Colombia |
| 10-TADO | Colombia | Choco | Tado | - | 5.2658 | -76.5647 | on-farm Colombia |
| 297 | Colombia | Guainia | Inirida | Panjuil - Tribu Puinabi | 3.8652 | -67.9263 | INIA |
| 340 | Colombia | Guainia | Inirida | Rio Guaviare - Jejen | 3.8652 | -67.9263 | INIA |
| 298 | Colombia | Guainia | Puerto Inirida | Panjuil - Tribu Puinabi | 3.8652 | -67.9263 | INIA |
| 14-ARMENIA | Colombia | Quindio | Armenia | - | 4.5000 | -75.7000 | on-farm Colombia |
| 15-ARMENIA | Colombia | Quindio | Armenia | - | 4.5000 | -75.7000 | on-farm Colombia |
| 16-ARMENIA | Colombia | Quindio | Armenia | - | 4.5000 | -75.7000 | on-farm Colombia |
| 19-BVTURA | Colombia | Valle del Cauca | Buenaventura | - | 3.8933 | -77.0697 | on-farm Colombia |
| 20-BVTURA | Colombia | Valle del Cauca | Buenaventura | - | 3.8933 | -77.0697 | on-farm Colombia |
| 12739-2 | Colombia | Valle del Cauca | Cali | - | 3.4469 | -76.5163 | CATIE |
| 12750-3 | Colombia | Valle del Cauca | Cali | - | 3.4469 | -76.5163 | CATIE |
| 12768-1 | Colombia | Valle del Cauca | Cali | - | 3.4469 | -76.5163 | CATIE |
| 12801-1 | Colombia | Valle del Cauca | Cali | - | 3.4469 | -76.5163 | CATIE |
| 9646-3 | Colombia | Valle del Cauca | Cali | - | 3.4469 | -76.5163 | CATIE |
| 9692-1 | Colombia | Valle del Cauca | Cali | - | 3.4469 | -76.5163 | CATIE |
| 9748-2 | Colombia | Valle del Cauca | Cali | - | 3.4469 | -76.5163 | CATIE |
| 9757-1 | Colombia | Valle del Cauca | Cali | - | 3.4469 | -76.5163 | CATIE |
| 289 | Colombia | Vaupes | Mitu | Arara | 1.2500 | -70.2166 | INIA |
| 285 | Colombia | Vaupes | Mitu | Piramiri | 1.2500 | -70.2166 | INIA |
| 329 | Colombia | Vaupes | Piracuara | Tribu Indigena Tucano | 4.0167 | -69.6500 | INIA |
| 330 | Colombia | Vaupes | Piracuara | Rio Papuri | 4.0167 | -69.6500 | INIA |
| 333 | Colombia | Vaupes | Piracuara | Rio Papuri - Caño Cabiyu | 4.0167 | -69.6500 | INIA |
| 12239-3 | Colombia |  | - | - | 4.0000 | -72.0000 | CATIE |
| 14142-6 | Costa Rica | Cartago | Jiménez | Oriente | 9.8333 | -83.6833 | CATIE |
| 14144-2 | Costa Rica | Cartago | Jiménez | Oriente | 9.8333 | -83.6833 | CATIE |
| 14150-19 | Costa Rica | Cartago | Jiménez | Oriente | 9.8333 | -83.6833 | CATIE |
| 14151-19 | Costa Rica | Cartago | Jiménez | Oriente | 9.8333 | -83.6833 | CATIE |
| 10002-2 | Costa Rica | Limón | Potocí | Guapiles | 10.2166 | -83.7833 | CATIE |
| 9983-1 | Costa Rica | Limón | Potocí | Guápiles | 10.0000 | -83.2167 | CATIE |
| 11326-4 | Costa Rica | Limón | Siquirres | - | 10.1000 | -83.5166 | CATIE |
| 7399-2 | Costa Rica | San Jose | San Isidro del General | Pérez Zeledón | 9.6667 | -84.0000 | CATIE |
| 255 | Ecuador | Napo | - | Carret. Petrolera Parker | -0.6667 | -77.8333 | INIA |
| 248 | Ecuador | Napo | Archidona | Rio Napo - Puerto Colon | -0.9166 | -77.8000 | INIA |
| 194 | Ecuador | Napo | Coca | Coca - Lago Agrio Km. 6 | -0.2333 | -77.7166 | INIA |
| 198 | Ecuador | Napo | Lago Agrio | Km. 20 Via Chuchufindi | 0.1000 | -76.8667 | INIA |
| 190 | Ecuador | Napo | Tena | Archidona - Poroto Yacu | -0.9166 | -77.8000 | INIA |
| 192 | Ecuador | Napo | Tena | Archidona - Poroto Yacu | -0.9166 | -77.8000 | INIA |
| 243 | Ecuador | Napo | Tena | Palmera - Chonta Punta | -0.9833 | -77.8166 | INIA |
| 244 | Ecuador | Napo | Tena | Palmera - Chonta Punta | -0.9833 | -77.8166 | INIA |
| 240 | Ecuador | Napo | Tena | Misavalle - Chinchipino | -0.9833 | -77.8166 | INIA |
| 346 | Ecuador | Pastaza | - | Caserio La Esperanza | -1.5833 | -77.7500 | INIA |
| 238 | Ecuador | Pastaza | Morona Santiago | Sucua | -2.4666 | -78.1666 | INIA |
| 11927-1 | Panamá | Colón | - | - | 9.1667 | -80.0000 | CATIE |
| 11929-13 | Panamá | Panamá | - | Lago Gatún | 9.2000 | -79.9167 | CATIE |
| 11930-13 | Panamá | Panamá | - | Lago Gatún | 9.2000 | -79.9167 | CATIE |
| 11931-11 | Panamá | Panamá | - | Lago Gatún | 9.2000 | -79.9167 | CATIE |
| 11934-2 | Panamá | Panamá | - | Lago Gatún | 9.2000 | -79.9167 | CATIE |
| 12236-2 | Panamá | Panamá | - | Lago Gatún | 9.2000 | -79.9167 | CATIE |
| 11926-1 | Panamá | Panamá | - | Capira | 8.7500 | -79.8833 | CATIE |
| 14467-2 | Peru | Loreto | Iquitos | - | -5.0000 | -75.0000 | CATIE |
| 14469-6 | Peru | Loreto | Iquitos | - | -5.0000 | -75.0000 | CATIE |
| 7596-2 | Peru | Loreto | Iquitos | - | -5.0000 | -75.0000 | CATIE |
| 171 | Peru | Loreto | Mazan | Mazan, Rio Napo | -3.4666 | -72.7500 | INIA |
| 172 | Peru | Loreto | Mazan | Mazan, Rio Napo | -3.4666 | -72.7500 | INIA |
| 174 | Peru | Loreto | Mazan | Mazan, Rio Napo | -3.4666 | -72.7500 | INIA |
| 177 | Peru | Loreto | Mazan | Mesopotamia, Rio Napo | -3.4666 | -72.7500 | INIA |
| 203 | Peru | Loreto | Tigre | Intuto | -3.5333 | -74.7333 | INIA |
| 204 | Peru | Loreto | Tigre | Intuto - Quebrada Intuto | -3.6500 | -74.7500 | INIA |
| 208 | Peru | Loreto | Tigre | Intuto - Quebrada Intuto | -3.6500 | -74.7500 | INIA |
| 213 | Peru | Loreto | Tigre | Intuto - Caserio 28 De Julio | -3.5333 | -74.7333 | INIA |
| 220 | Peru | Loreto | Tigre | Intuto - Caserio A. Ugarte | -3.5333 | -74.7333 | INIA |
| 221 | Peru | Loreto | Tigre | Intuto | -3.5333 | -74.7333 | INIA |
| 14508-2 | Perú | Loreto | Alto Amazonas | Yurimaguas | -5.9000 | -76.0833 | CATIE |
| 14517-2* | Perú | Loreto | Alto Amazonas | Yurimaguas | -5.9000 | -76.0833 | CATIE |
| 12719-2 | Perú | Loreto | Yurimaguas | - | -5.9000 | -76.0833 | CATIE |
| 12729-4 | Perú | Loreto | Yurimaguas | - | -5.9000 | -76.0833 | CATIE |
| 12733-4 | Perú | Loreto | Yurimaguas | - | -5.9000 | -76.0833 | CATIE |
| 14538-1* | Perú | Madre de Dios | Puerto Maldonado | - | -12.6000 | -69.1833 | CATIE |
| 251-8 | Peru | Loreto | Tigre | Intuto - Quebrada Intuto | -3.6500 | -74.7500 | INIA |
