# Supplementary material for: An Integrated Hypothesis on the Domestication of Bactris gasipaes
Source: PLoS One. 2015 Dec 10;10(12):e0144644. doi: 10.1371/journal.pone.0144644 (PMC4675520; doi:10.1371/journal.pone.0144644)
Supplement: S2 Table — (DOCX) [file pone.0144644.s005.docx]

| **Locus** |  | **Primer sequence 5'-3'** | **Expected product (bp)** | **No of alleles** |
| --- | --- | --- | --- | --- |
|  |  |  |  |  |
| **BG-1** | **F:** | TTTCCTTCCGCAATACAAT | 151 | 6 |
|  | **R:** | CACCAATCCTCTGTGCAA |  |  |
| **BG-9** | **F:** | TCCGATTGTAGAGACCGACC | 201 | 7 |
|  | **R:** | CAGGTTGCTCCTGTTGGTAAC |  |  |
| **BG-11** | **F:** | AGAGGAGGAAAGCAAGCGT | 241 | 13 |
|  | **R:** | CGACCATTCAGTCCATCATC |  |  |
| **BG-17** | **F:** | TTGTCTGCTCTAGCTCATTTGG | 228 | 7 |
|  | **R:** | CGCTCAATCCAGTGCAAG |  |  |
| **BG-24** | **F:** | ACGTAAGGTTGAATGCCTGC | 256 | 8 |
|  | **R:** | TCTGCAACTCTCATGGTGATTC |  |  |
| **BG-44** | **F:** | AGCCGAGGAAATATGATGG | 166 | 7 |
|  | **R:** | AGCTACAATCGCTCTCCG |  |  |
| **BG-51** | **F:** | CCTGGTTGTTTTAGATACTGTTGG | 169 | 9 |
|  | **R:** | TTCAGTGCAAACCTTATTGACA |  |  |
| **BG-55** | **F:** | TTCTGGGTGCGGTGGTAG | 278 | 6 |
|  | **R:** | ATGATGGACTGAAGAGATGGAATAG |  |  |
| **BG-63** | **F:** | AAGCAAGGAAAGCCATCG | 128 | 9 |
|  | **R:** | TATCTCCAGTAGCCCAGGTG |  |  |
