# Supplementary material for: An Integrated Hypothesis on the Domestication of Bactris gasipaes
Source: PLoS One. 2015 Dec 10;10(12):e0144644. doi: 10.1371/journal.pone.0144644 (PMC4675520; doi:10.1371/journal.pone.0144644)
Supplement: S3 Table — (DOCX) [file pone.0144644.s006.docx]

|  |  | **Correlation method** | **R** | **t** | **p** | **df** |
| --- | --- | --- | --- | --- | --- | --- |
| **Richness** | 3-6 trees | Pearson | 0.97 | 99.03 | < 2.2e-16 | 530 |
|  | 3-10 trees | Pearson | 0.90 | 21.27 | < 2.2e-16 | 106 |
| **Locally common alleles** | 3-6 trees | Pearson | 0.98 | 120.78 | < 2.2e-16 | 530 |
|  | 3-10 trees | Pearson | 0.88 | 18.64 | < 2.2e-16 | 106 |
