# Supplementary material for: An Integrated Hypothesis on the Domestication of Bactris gasipaes
Source: PLoS One. 2015 Dec 10;10(12):e0144644. doi: 10.1371/journal.pone.0144644 (PMC4675520; doi:10.1371/journal.pone.0144644)
Supplement: S4 Table — Allele number, average allele frequency, observed and expected heterozygosity, and the fixation index Fis were measured on Bactris gasipaes var. gasipaes accessions from CATIE, INIA and those collected on-farm in Colombia. (DOCX) [file pone.0144644.s007.docx]

| **Locus ID** | **No. alleles** | **Avg allele freq** | **Ho** | **He** | **Fis** |
| --- | --- | --- | --- | --- | --- |
| BG-1 | 12 | 0.083 | 0.641 | 0.873 | 0.272 |
| BG-9 | 7 | 0.142 | 0.558 | 0.831 | 0.334 |
| BG-11 | 6 | 0.167 | 0.688 | 0.710 | 0.037 |
| BG-17 | 5 | 0.200 | 0.350 | 0.636 | 0.455 |
| BG-24 | 4 | 0.250 | 0.525 | 0.659 | 0.209 |
| BG-44 | 8 | 0.125 | 0.481 | 0.684 | 0.303 |
| BG-51 | 9 | 0.111 | 0.538 | 0.857 | 0.378 |
| BG-55 | 5 | 0.200 | 0.488 | 0.639 | 0.243 |
| BG-63 | 12 | 0.083 | 0.750 | 0.881 | 0.154 |
| Average | 7.556 | 0.1493 | 0.557 | 0.752 | 0.265 |
