# Supplementary material for: An Integrated Hypothesis on the Domestication of Bactris gasipaes
Source: PLoS One. 2015 Dec 10;10(12):e0144644. doi: 10.1371/journal.pone.0144644 (PMC4675520; doi:10.1371/journal.pone.0144644)
Supplement: S1 Text — (DOCX) [file pone.0144644.s008.docx]

DNA extraction was conducted according to the protocol developed by Afanador and Haley [76] and its quantity and quality verified through electrophoresis on 1% agarose gels stained with SYBR Safe (DNA gel stain – Invitrogen). PCR reactions were conducted in a PTC-100TM thermocycler (Programmable Thermal Controller MJ Research, Inc.). PCR reactions were performed in a total volume of 25 μl, with 5 μl of genomic DNA (4 ng/μl), 2 μl Buffer 1X (100mM Tris HCl, 500mM KCl, 1% triton X-100), magnesium chloride (MgCl) 1.5 mM, dNTPs 0.25 mM, 0.75 μl primers, 0.37mM and 0.2 μl Taq polimerase CIAT (2:1). The PCR cycle was as follows: 35 thermal cycles at 95 ⁰C for 2 minutes, 50-55 ⁰C (depending on the primers’ annhealing temperature) for 45 seconds, 72 ⁰C for a minute and a half and a final extension at 72 ⁰C for a minute and a half. PCR products were visualized on 6 % bisacrylamide gels and interpreted visually through silver staining.
