# Supplementary material for: An Integrated Hypothesis on the Domestication of Bactris gasipaes
Source: PLoS One. 2015 Dec 10;10(12):e0144644. doi: 10.1371/journal.pone.0144644 (PMC4675520; doi:10.1371/journal.pone.0144644)
Supplement: S2 Text — (DOCX) [file pone.0144644.s009.docx]

Fruits were photographed and their weight and dimensions (length and width) were measured. Dry matter was obtained by placing ground fruit samples in an oven at 40 ^0^C for 48 hours. HPLC analyses were carried out on both raw and cooked fruit pulp to identify and quantify carotenoids [78]. Fruits were then ground to flour for subsequent measurements of protein, ash, fiber and oil content. Ash, which provides a measure of the total amount of minerals within a food, was measured by incinerating the flour obtained from the fruits in an oven at 550 ⁰C for 3 hours. Raw fibre content was determined by combustion of the dry matter residue of the flour samples, after a digestion with acids and base reagents. Nitrogen content was quantified in a colorimeter (660 nm wavelength) after digesting the fruit flour with sulfuric acid, selenium and salicylic acid; protein content was estimated multiplying the nitrogen content by a factor of 5.6 [79]. Oil content was determined by extraction from the flour with hexane [80]. Starch was isolated from the flour and analysed by RVA (Rapid Visco Analyser). Starch functional properties were determined by a RVA (Rapid Visco Analyzer) on a 10% starch suspension. The temperature profile used started at 50 ⁰C, increased to 93 ⁰C (6 °C/minute), maintained the temperature for 5 minutes and then decreased to 50 ⁰C again (6 ⁰C/minute). Pasting temperature and maximum viscosity were recorded.
