# Supplementary material for: An Integrated Hypothesis on the Domestication of Bactris gasipaes
Source: PLoS One. 2015 Dec 10;10(12):e0144644. doi: 10.1371/journal.pone.0144644 (PMC4675520; doi:10.1371/journal.pone.0144644)
Supplement: S3 Text — Models considered and calibration methods. (DOCX) [file pone.0144644.s010.docx]

Modeling algorithms considered were maximum entropy (MAXENT), boosted regression trees (BRT; including stepwise selection of explanatory variables), random forests (RF), generalized linear models (GLM, including stepwise selection of explanatory variables), generalized additive models (GAM, including stepwise selection of explanatory variables), multivariate adaptive regression splines (MARS), regression trees (RT), artificial neural networks (ANN), flexible discriminant analysis (FDA), support vector machines (SVM), and the BIOCLIM algorithm. Background points (an overall maximum of 20,000 and maximum one per grid cell) were randomly selected from the area enclosed by a convex hull polygon constructed around all presence points and extended with a buffer corresponding to 10% of the polygon’s largest axis. Modeling was performed at 2.5′ spatial resolution and we retained only one presence point per grid cell. Models for projection to past climate conditions were calibrated based on current climate data obtained from the Worldclim database (i.e. averages from 1950–2000)[81]. For projection to future climate scenarios we added the following variables to the calibration layers: ecoregions [82], soil types [83], aspect, slope, terrain roughness index, and the direction of water flow. Collinear environmental layers were removed based on iterative calculations of variance inflation factors (VIF), retaining only variables with VIFs smaller than 5. The retained variables used in model calibration for posterior projection to past climate conditions were bio2, bio3, bio4, bio5, bio7, bio8, bio13, bio15, bio18 and bio19, and for projection to future climate bio2, bio3, bio4, bio5, bio7, bio13, bio15, bio18, bio19 (all explained in the box below), slope, aspect, direction of water flow, terrain roughness index, soil types and ecoregions.

| BIO2 = Mean Diurnal temperature Range (Mean of monthly (max temp - min temp))  BIO3 = Isothermality (BIO2/BIO7) (* 100)  BIO4 = Temperature Seasonality (standard deviation *100)  BIO5 = Max Temperature of Warmest Month  BIO7 = Temperature Annual Range (BIO5-BIO6)  BIO8 = Mean Temperature of Wettest Quarter  BIO13 = Precipitation of Wettest Month  BIO15 = Precipitation Seasonality (Coefficient of Variation)  BIO18 = Precipitation of Warmest Quarter  BIO19 = Precipitation of Coldest Quarter |
| --- |
